# Electrochemical biosensing in oncology: a review advancements and prospects for cancer diagnosis

**DOI:** 10.1080/15384047.2025.2475581

**Published:** 2025-03-13

**Authors:** Sana Noreen, Izwa Ishaq, Muhammad Hamzah Saleem, Baber Ali, Syed Muhammad Ali, Javed Iqbal

**Affiliations:** a University Institute of Diet and Nutritional Sciences, The University of Lahore, Lahore, Pakistan; b College of Plant Science and Technology, Huazhong Agricultural University, Wuhan, China; c Department of Plant Sciences, Quaid-i-Azam University, Islamabad, Pakistan; d Nursing Department, Communicable Disease Center Hamad Medical Corporation, Doha, Qatar; e Department of Surgery, Hamad Medical Corporation, Doha, Qatar

**Keywords:** Electrochemical biosensing, cancer diagnostics, biomarkers, electrode design, oncology, microfluidics, point-of-care testing

## Abstract

Early and precise diagnosis of cancer is pivotal for effective therapeutic intervention. Traditional diagnostic methods, despite their reliability, often face limitations such as invasiveness, high costs, labor-intensive procedures, extended processing times, and reduced sensitivity for early-stage detection. Electrochemical biosensing is a revolutionary method that provides rapid, cost-effective, and highly sensitive detection of cancer biomarkers. This review discusses the use of electrochemical detection in biosensors to provide real-time insights into disease-specific molecular interactions, focusing on target recognition and signal generation mechanisms. Furthermore, the superior efficacy of electrochemical biosensors compared to conventional techniques is explored, particularly in their ability to detect cancer biomarkers with enhanced specificity and sensitivity. Advancements in electrode materials and nanostructured designs, integrating nanotechnology, microfluidics, and artificial intelligence, have the potential to overcome biological interferences and scale for clinical use. Research and innovation in oncology diagnostics hold potential for personalized medicine, despite challenges in commercial viability and real-world application.

## 1. Introduction

### 1.1. What is cancer and its global impact

Cancer is a term used to describe a collection of related diseases and is renowned for being the second most deadly disease globally, following cardiovascular disease in terms of mortality.^1^ Cancer is a multifaceted and multistage disease characterized by a wide range of genetic and epigenetic alterations that disrupt normal cellular processes. These alterations interfere with cellular signaling pathways, leading to the transformation of normal cells into malignant tumor-causing cells. Under normal circumstances, human cells divide to create new cells as needed, while old or damaged cells undergo programmed cell death and are replaced by healthy ones.^2^ However, cancer disrupts this orderly process; old or injured cells survive, and new abnormal cells are produced. These abnormal cells divide uncontrollably, potentially forming tumors and invading neighboring tissues.^3^

Cancer manifests in various forms, including breast, skin, lung, colon, prostate, and lymphoma, with each type presenting distinct symptoms, diagnostic challenges, and treatment options.^4^ Both environmental and genetic factors contribute to cancer development, with known risk factors including radiation, chemical exposure, tobacco use, alcohol consumption, and immune dysfunction.^5^ Additionally, infections caused by certain bacteria and viruses, such as Helicobacter pylori and human papillomavirus, can lead to stomach and cervical cancers, respectively.^6^ Globally, cancer poses a vast and multifaceted challenge. According to the World Health Organization, it is one of the leading causes of death worldwide, with millions of new cases diagnosed annually. Beyond its devastating impact on human lives, cancer also imposes a significant economic burden, with billions spent each year on care and treatment.^7^ Moreover, the emotional and psychological toll on patients and their families is incalculable.^8^ Advances in modern molecular techniques have enhanced our understanding of cancer and provided new opportunities to identify genomic and proteomic biomarkers.^9^ These developments are crucial for achieving early detection and effective treatment, which are essential in combating the disease.

**Figure 1.**
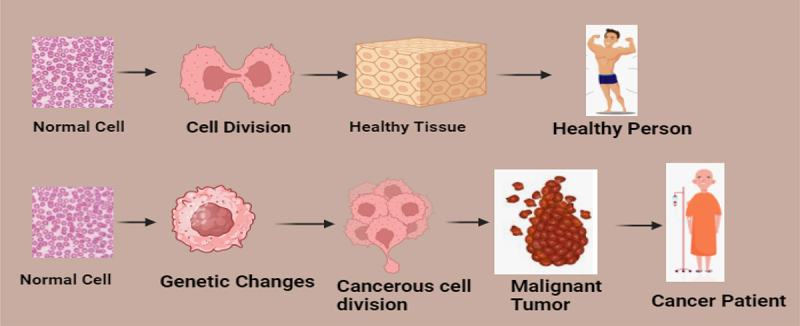
Progression from Normal Cells to cancer Cells and its impact on health. The upper pathway illustrates the normal process of cell division, where healthy cells divide to form tissues that maintain proper bodily functions, ensuring the overall health of an individual. The lower pathway demonstrates the development of cancer, starting with genetic changes in normal cells. These alterations lead to uncontrolled and abnormal cell division, forming a malignant tumor. As the tumor progresses, it disrupts normal bodily functions, ultimately manifesting in the condition of a cancer patient requiring medical intervention. This figure highlights the stark contrast between normal cellular processes and cancerous transformations, emphasizing the importance of early detection and intervention in cancer care.

Innovative diagnostic approaches, including lab-on-a-chip and point-of-care (POC) devices, are addressing the challenges associated with traditional cancer detection methods.^10^ Such technologies enable multi-analyte analysis and early identification of minute irregularities in biomarkers, facilitating timely diagnosis and personalized treatment^11^ (Figure 1). For example, multicolor quantum dots (QDs) have proven to be highly effective in medical imaging, capable of detecting tens to hundreds of cancer biomarkers in blood assays.^12^ These advancements underscore the importance of early and accurate cancer diagnosis in improving patient outcomes and reducing the global burden of this deadly disease.^13^

### 1.2. The necessity for early and accurate diagnosis

The importance of early and accurate diagnosis in the context of cancer cannot be overstated. Detecting cancer at an early stage increases the chances of successful treatment and reduces the risk of mortality. Early diagnosis means the cancer might be localized and hasn’t spread, making treatments more effective and increasing the chances of recovery.^14^

However, the symptoms of cancer can be nonspecific and similar to other diseases. Therefore, accurate diagnostic tools are crucial to differentiate cancer from other conditions and to determine the type and stage of cancer. The right diagnosis ensures the patient receives the most appropriate and effective treatment. Moreover, an accurate diagnosis informs the prognosis – the likely course and outcome of the disease. Knowing the specifics of the cancer can help healthcare professionals design a tailored treatment plan and counsel patients on what to expect and how to manage the disease.

### 1.3. An introduction to various diagnostic methods

Diagnosing cancer is a complex process that may involve multiple methods. The method employed often depends on the suspected type of cancer, the patient’s medical history, and the symptoms they are experiencing.^15^

Physical examinations can sometimes reveal signs of certain types of cancers. For instance, skin cancer can often be detected during a routine skin examination, or a doctor might feel a suspicious lump during a breast or prostate exam.^16^ Imaging tests like X-rays, CT scans, MRI scans, and PET scans can create pictures of the inside of the body and may reveal tumors. However, imaging tests alone can’t confirm if a growth is cancerous.^17^ An under-the-microscope tissue sample biopsy is one of the most accurate cancer diagnosis. Cell appearance distinguishes benign and malignant tumors. A blood test may also disclose something. They can’t identify cancer, but liver and kidney function tests can indicate a malignancy.

#### 1.3.1. Advantages of traditional cancer screening

Traditional cancer screening methods offer several significant benefits that make them essential tools in the early detection and management of cancer. One of the primary advantages is the ability to detect cancer at an early stage when it is more likely to respond to treatment effectively and is less likely to metastasize. Early detection can significantly reduce the complexity and intensity of required treatments, potentially leading to shorter recovery periods and less invasive interventions. Another critical benefit of early screening is the improved prognosis for patients, as it increases the likelihood of survival by identifying cancer before it progresses to an advanced stage. Traditional screening also allows for the initiation of treatment even before clinical symptoms become apparent, which can be pivotal in managing certain types of cancer. Furthermore, for individuals undergoing regular screening, a “normal result” (i.e., a result indicating no signs of cancer or abnormalities) can provide reassurance and psychological relief, reducing anxiety about their health status. This reassurance can encourage adherence to routine screening programs, further enhancing the likelihood of early detection in the future. Despite its challenges, traditional cancer screening remains a cornerstone in oncology care, offering substantial benefits in improving outcomes and quality of life for patients.^18^

#### 1.3.2. Limitations of traditional cancer screening

Traditional cancer screening methods, while valuable, have several limitations. False-positive test results can incorrectly indicate the presence of cancer, leading to unnecessary stress and additional testing. Conversely, false-negative results, though rare, may fail to detect existing cancer, delaying treatment and potentially worsening outcomes. Some screening procedures can be uncomfortable and may require further invasive diagnostic tests, which can be physically and emotionally taxing for patients. Overdiagnosis, where non-threatening or slow-growing cancers are detected, can result in unwarranted anxiety and unnecessary medical interventions. Certain screening tests also carry inherent risks; for example, colon cancer screening via colonoscopy may, in rare cases, cause tears in the colon lining. Furthermore, the financial cost of these screening techniques can be prohibitive for some individuals, limiting their accessibility and widespread use.

### 1.4. The importance of electrochemical methods

Electrochemical methods have emerged as a transformative tool in cancer diagnostics due to their ability to measure electrical signals generated by chemical reactions. These reactions occur at the interface of an electrode and a biological sample, where the transfer of electrons provides valuable information about the presence and concentration of specific cancer biomarkers. These biomarkers include proteins, nucleic acids, and metabolites that are uniquely expressed or elevated in certain types of cancer.^19^ The unique advantages of electrochemical methods lie in their speed, cost-effectiveness, and exceptional sensitivity, which often surpass those of traditional diagnostic techniques. These methods are highly adaptable to miniaturization, allowing for the development of portable, point-of-care diagnostic devices that can bring cancer screening closer to patients. Additionally, the ability to integrate electrochemical platforms with advanced technologies such as microfluidics and nanotechnology further enhances their potential for rapid and precise detection.^20^

Despite these advantages, several challenges and limitations persist. For instance, the performance of electrochemical biosensors can be influenced by biological interferences and variability in sample matrices. Furthermore, current knowledge about the long-term stability, reproducibility, and scalability of these methods remains limited, hindering their widespread clinical application. Addressing these controversies is crucial for optimizing the clinical utility of electrochemical methods and underscores the need for continued research in this domain. By addressing these limitations and advancing the understanding of electrochemical methods, this field holds the promise to revolutionize cancer diagnostics, offering a future where early and precise detection is accessible to all.

## 2. Basics of electrochemical methods

### 2.1. Introduction to electrochemistry and its relevance in biological applications

Electrochemistry can be defined as the branch of chemistry that deals with the relationship between electrical energy and chemical change. At its core, it’s about understanding how electrons move between atoms and molecules, and how this movement relates to the broader behaviors of chemical systems. Electrochemical processes are redox reactions where electrons are transferred from one species to another.^21^ Biological systems are rife with examples of redox processes. From the fundamental reactions that provide energy to cells, such as cellular respiration, to the transmission of electrical impulses along nerve cells, the interplay between electricity and chemistry is a foundational aspect of life.

Given this inherent electrochemical nature of biological systems, it’s only logical that electrochemistry would find applications in biological research and clinical diagnostics. These applications allow researchers and clinicians to investigate and measure biological phenomena with precision, providing insights that might be difficult to achieve through other means.^22^

### 2.2. Principle of electrochemical detection

The underlying principle of electrochemical detection is relatively straightforward: when a chemical reaction occurs that involves the transfer of electrons (a redox reaction), it’s possible to measure the associated electrical current. This current can provide quantitative information about the concentration of the involved species.^23^ In an electrochemical cell, there are typically two electrodes: an anode and a cathode. When a potential (voltage) is applied across these electrodes, it drives a redox reaction. At the anode, oxidation occurs (loss of electrons), while at the cathode, reduction takes place (gain of electrons). By monitoring the current that flows in response to the applied potential, it’s possible to gather information about the reaction and, by extension, about the system under study.^24^ For instance, if a molecule of interest (like a cancer biomarker) undergoes a redox reaction under certain conditions, the resulting current can be measured. The magnitude of this current can then be correlated with the concentration of the molecule, providing a quantitative measure.^25^

### 2.3. Main types of electrochemical techniques

Electrochemical techniques have evolved over the years, with various methods being developed to cater to specific applications and research questions. A brief overview of some primary techniques follows:

#### 2.3.1. Voltammetry

In voltammetry, the potential (or voltage) applied to the working electrode is varied in a controlled manner, and the resulting current is measured. The resulting curve (current vs. potential) provides information about the redox processes occurring at the electrode surface. The shape and position of peaks in this curve can give clues about the identity and concentration of electroactive species.^26,27^

#### 2.3.2. Amperometry

**Figure 2.**
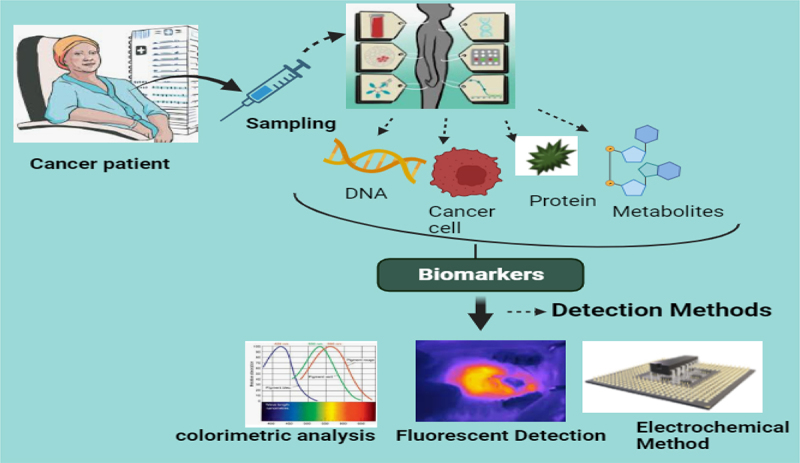
Overview of biomarker detection methods for cancer diagnostics. The figure illustrates the process of cancer biomarker detection starting with the sampling stage, where biological samples are collected from cancer patients. These samples may include DNA, cancer cells, proteins, or metabolites that serve as biomarkers for disease identification. The biomarkers are subsequently analyzed using advanced detection methods, including colorimetric analysis, fluorescent detection, and electrochemical methods. Each detection technique offers unique advantages in terms of sensitivity, specificity, and application, contributing to the accurate and early diagnosis of cancer. The integration of these methods represents a critical step in advancing personalized cancer care and improving patient outcomes.

Amperometric biosensors measure an analytical’s concentration at a fixed voltage and return the current in response. Analyzing the produced current indicates that the analyte is engaged in a redox process. In amperometry, the working electrode is subjected to a constant potential, and the resultant current is measured. The concentration of the electroactive species is directly correlated with the current. When a quick and continuous reading is needed, like in real-time monitoring, this technique comes in handy.^28^ There are, of course, other electrochemical techniques and variations of the above, like cyclic voltammetry, impedance spectroscopy, and potentiostatic methods. Each has its unique advantages and best-fit applications, but all share the foundational principle of leveraging the relationship between electrical and chemical behavior to gather valuable information.^29^ In summary, electrochemical methods harness the innate redox behaviors present in chemical and biological systems to provide powerful diagnostic and analytical tools. Their sensitivity, speed, and adaptability make them particularly well-suited for applications in areas like cancer diagnostics, where timely and accurate detection is of paramount importance as shown in Figure 2. Fortunately, the fixed potential during amperometric detection reduces the background signal that negatively impacts the limit of detection by producing a small charging current (the current required to apply the potential to the system). Furthermore, considerably improved transportation of mass to the electrode surface can be obtained using hydrodynamic amperometric approaches.

## 3. Advantages of electrochemical methods in cancer diagnosis

### 3.1. Sensitivity and specificity

One of the primary strengths of electrochemical methods in cancer diagnosis lies in their inherent sensitivity. These methods can detect minute concentrations of specific molecules, often down to the nanomolar or even picomolar range. This high sensitivity ensures that even trace amounts of cancer biomarkers can be detected, making early diagnosis a possibility.^19^ In addition to sensitivity, electrochemical methods also offer high specificity. Through the design of specific probes or the choice of appropriate electrochemical conditions, these methods can distinguish between closely related molecules. In the context of cancer diagnosis, this means that specific types or stages of cancer can potentially be identified based on unique biomarker profiles.

### 3.2. Potential for miniaturization and portability

Electrochemical methods lend themselves well to miniaturization. Modern electrode designs, often utilizing materials like gold, carbon nanotubes, or graphene, can be manufactured at a micro or nano scale. Such miniaturized systems are not only efficient but also portable. Portability is a game-changer in healthcare diagnostics. A portable diagnostic device means that testing can be conducted outside traditional laboratory settings – in clinics, at patients’ homes, or in remote areas with limited healthcare infrastructure. For patients, this could translate to faster results and timely interventions.

### 3.3. Cost-effectiveness and potential for rapid diagnosis

Traditional cancer diagnostic methods can be expensive, often requiring specialized equipment and extended laboratory processing times. Electrochemical methods, in contrast, are generally more cost-effective. The materials and equipment, especially when miniaturized, can be produced at a reduced cost. Furthermore, the rapid nature of electrochemical reactions means that diagnostic results can often be obtained in real-time or within a few minutes, eliminating the waiting period typical of many other diagnostic approaches.

### 3.4. Compatibility with microfluidic systems and potential for point-of-care testing

Electrochemical methods are remarkably compatible with microfluidic systems. Microfluidics involves the manipulation of small amounts of fluids in channels with dimensions of tens to hundreds of micrometers. By integrating electrochemical detection within microfluidic devices, it’s possible to create highly efficient, compact systems that can process and analyze small samples with precision.^30^

Such integration holds immense potential for point-of-care (POC) testing. POC devices aim to bring diagnostic tests directly to the patient, often providing results during a single visit or interaction. When combined with the sensitivity and specificity of electrochemical methods, POC devices can offer timely and accurate cancer diagnoses, leading to faster treatment decisions and better patient outcomes. In conclusion, the advantages of electrochemical methods in cancer diagnosis are manifold. From their inherent sensitivity and specificity to the possibilities they offer for portability, rapid results, and point-of-care testing, these methods represent a promising frontier in the ongoing battle against cancer.^31^

## 4. Electrochemical biosensors in cancer diagnosis

### 4.1. Introduction to biosensors

**Figure 3.**
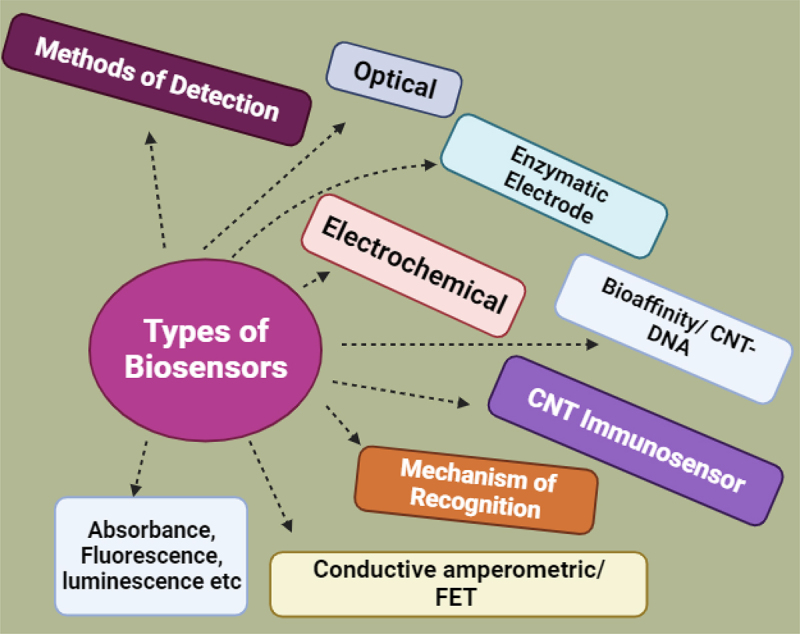
Classification and detection mechanisms of biosensors. The figure categorizes the different types of biosensors, highlighting their methods of detection and mechanisms of recognition. Key biosensor types include optical sensors, electrochemical sensors, and carbon nanotube (cnt)-based immunosensors. Detection methods encompass absorbance, fluorescence, and luminescence techniques. The mechanisms of recognition involve enzymatic electrodes, bioaffinity interactions (e.g., CNT-DNA), and conductive amperometric or field-effect transistor (FET) approaches. This framework illustrates the diverse strategies employed by biosensors to achieve high sensitivity and specificity in detecting biological and chemical analytes, particularly in cancer diagnostics.

Biosensors are analytical devices that combine a biological component, like a protein, enzyme, or cell, with a transducer to detect specific biochemical reactions and produce a measurable signal. This fusion of biological specificity with analytical sensitivity allows biosensors to detect and quantify a wide range of compounds, from simple ions to complex molecules and cells.^5^ Biosensors advanced research at a new level. Biosensing is the absence of set techniques for producing an accessible detection signal of interaction between biological molecules. Biosensors detect these molecular interactions.^32^ Many classification techniques have been developed for biosensors; however, the most widely applied scheme is based on two factors: signal transduction and the biorecognition component. Optical sensors are by far among the most common biosensors, followed by electrochemical systems. Very High responsiveness, durability, speed of detection, and capacity to identify numerous analytes are characteristics of these.^33^ Based on how signals are transduced, biosensors can be divided into four groups: mass sensitive, thermal, optical, and electrochemical sensors. Electrochemical biosensors are compatible with contemporary microfabrication processes, highly sensitive, portable, and reasonably priced. Biosensors can be categorized as enzymatic, protein receptor-based, immunosensors, DNA biosensors, or whole-cell biosensors based on the biological recognition element. Biosensors are useful in many domains, but one of the most important ones is cancer diagnosis because of the intricate and exact nature of many biological responses. As shown in Figure 3, there are several kinds of biosensors that are used to identify cancer at different stages.

Tracking proteins on tumor cell membranes and/or cancer-associated microRNA may provide biomarkers. Electrochemical methods are recommended for biomarker diagnosis because to their cheap cost, fast responsiveness, simplicity of operation, quantifiability, miniaturization potential, high sensitivity and selectivity, and reduced detection limit. One biorecognition element, one signal transducer, and three electrochemical systems make up electrochemical biosensors. Electrochemical reactions with target components on the electrode surface are recorded as the electrical signal changes. They created biorecognition components to identify cancer biomarkers. These include antibodies, enzymes, aptamers, DNA fragments, and peptides.^25,34^ Based on biorecognition components, biosensors are immunosensors, aptasensors, enzymatic biosensors, and Geno biosensors.

### 4.2. Types of biosensors used in cancer diagnosis

#### 4.2.1. DNA biosensors

These biosensors detect specific sequences of DNA, often related to oncogenes or tumor suppressor genes that might indicate a predisposition to certain cancers or confirm the presence of a cancerous mutation. A DNA biosensor typically has a probe – a short sequence of DNA that can hybridize (bind) to a target sequence. When the target sequence is present, binding occurs, leading to a measurable electrochemical change. The stability, specificity, and inexpensive cost of these biosensors make them alternatives to antibodies. DNA-aptamers-based biosensors can bind bacteria, viruses, proteins, hormones, analytes, tiny molecules, and ions with high specificity and affinity.^33,35^

#### 4.2.2. Protein biosensors

Protein biosensors use cell membrane protein receptors. These receptors allow metabotropic (enzyme secretion) or ionotropic receptors to transduce the binding signal across the membrane.^33^ Many cancers produce specific proteins or cause alterations in protein levels that can be indicative of the disease. Protein biosensors can detect these proteins at very low concentrations. They often use antibodies or aptamers as the biological component, as these molecules can bind to specific proteins with high affinity.

#### 4.2.3. Cell-based biosensors

Cell-based microbial biosensors detect chemical composition, toxicity, carcinogenicity, and mutagenicity in real time and cost-effectively using prokaryotic or eukaryotic cells.^36^ Cell-based microbial biosensors use prokaryotic or eukaryotic cells to detect chemical composition, toxicity, carcinogenicity, and mutagenicity in real time and cost-effective.^37^ Instead of detecting molecules, these biosensors detect changes in living cells that might be indicative of cancer. For instance, certain electrochemical changes might occur on the surface of cancerous cells, different from healthy cells. Cell-based biosensors can detect these changes.^38^ They are particularly valuable in assessing the overall health of a cell population, understanding cancer cell behaviors, or evaluating how cells respond to potential treatments as mentioned in Table 1.

**Table 1.** Types of biosensors use in assessing overall health of cell population.

Biosensors	Functions	References
Electrochemical biosensors	Electrochemical biosensors are mostly used to detect glucose levels, DNA-binding medicines, and hybridized DNA. They combine electrical and biological detection methods. These sensors work through an electrochemical interaction between the target analyte and the working electrode surface. When the analyte reacts with the working electrode, current, impedance, and potential are measured. Based on how they monitor electricity, electrochemical biosensors fall into three categories: (1) conductimetric, (2) amperometric, (3) potentiometric. Electrochemistry is better than optical approaches because analyzers may use turbid samples and the equipment is cheaper. Electrochemical methods are less selective and sensitive than optical approaches.	4 39 40
Multiplexed immune checkpoint biosensor (MICB)	Highly particular and discriminatory Small sample volumes are required for multiplex biosensors to diagnose the condition and identify several immunological checkpoints immediately. These sensors, the analyte tends to take longer to bind with the cell surface without the use of nanofluidic mixing. MICB has capability to evaluate 28 samples in less than two hours. MICB was evaluated for its capacity to detect soluble PD-1, PD-L1, and LAG-3 in liquid biopsies utilizing immune checkpoint concentrations in diluted human serum. Fortified samples had a concentration equal to the prognostic cut-off, which may differ from the cancer detection threshold, whereas immune checkpoint samples showed a different signal response.	41 42
Exosome Biosensor	Exosomes are essential for surface biomarker-mediated intercellular communication. For instance, exosomal proteins are essential for drug resistance, uniqueness, and the growth of cancer. This biosensor achieved optical resolution by means of laser beams. The positioning of the two laser beams, in essence, allowed SPR to interact with the exosomal proteins on the sensor’s surface. • The two photodetectors measured exosomal proteins by magnifying the original and reflection beams, using the other beam as a reference. Researchers evaluated biomarker specificity and sensitivity in serum samples from lung cancer patients using this biosensor.	42 43
Surface plasmon resonance (SPR) biosensor	When it depends on identifying PD-L1 in serum samples, including one from a patient with Stage III lung cancer, the SPR biosensor is incredibly precise and efficient. Evanescent field-based optical sensors, built with a thin gold layer, are effective for sensing. Photodetector array sensors find reflection minima in analyte flow across a gold-immobilized interactant. Through immunoreactions, SPR has identified illness microorganisms.	44 4 45
Optical biosensor	. A transducer system integrated with a biorecognition element makes up an optical biosensor, which is a small analytical instrument. An optical signal that optical biosensors produce is directly proportional to the analyte’s concentration. Biological and chemical compounds can be detected in real time, label-free, and with great sensitivity, specificity, and economy using optical biosensors. High specificity, sensitivity, compact size, and cost-effectiveness are some of the benefits.	46,47
Piezoelectric Sensors	These sensors respond rapidly and with highly effective sensitivity. A piezoelectric (PZ) sensor may detect minute changes in biorecognition element mass, frequency, or other quantitative attributes by attaching molecules to antibodies. The PZ sensor was developed to identify biomarkers for cervical cancer in concentration ranges of 50 to 1200 ng mL−1. Its analysis time was quite short. The marker can be found at concentrations 100 million times lower with a PZ sensor. In order to convey the biomarker’s existence, piezoelectric sensors require an adjustment in the sensor. PZ sensors are a dependable choice for efficient cancer detection since they are durable and sensitive to minuscule biomarker concentrations.	48 49 50

### 4.3. Mechanisms of target recognition and signal generation

The success of biosensors, particularly in cancer diagnosis, hinges on two primary mechanisms: target recognition and signal generation.

#### 4.3.1. Target recognition

This is the first step in biosensor functioning, where the biological component of the sensor (like DNA, protein, or cells) interacts with the target molecule or cell. This interaction is typically highly specific. For instance, in a DNA biosensor, the probe DNA sequence is chosen to perfectly match a target sequence, ensuring that only the intended target binds to the sensor.

#### 4.3.2. Signal generation

Once the target is recognized and bound, it triggers a change in the sensor. In electrochemical biosensors, this change typically involves a redox reaction that either produces or consumes electrons. The transducer component of the biosensor detects this change and converts it into an electrical signal. The magnitude and nature of this signal depend on the concentration and nature of the target, allowing for both qualitative (is the target present?) and quantitative (how much of the target is there?) analysis.

It’s worth noting that the signal’s accuracy and strength depend on several factors, including the quality of the biological recognition element, the efficiency of the transducer, and the absence of interferences or contaminants.

To wrap up, electrochemical biosensors in cancer diagnosis represent a convergence of biology and technology, marrying the specificity of biological reactions with the sensitivity of electrochemical detection. Whether it’s tracking DNA mutations, identifying specific proteins, or monitoring cell behaviors, these biosensors hold significant promise in advancing our ability to detect and understand cancer.

## 5. Recent advances in electrochemical detection of cancer biomarkers

### 5.1. Various cancer biomarkers detectable electrochemically

Cancer biomarkers, generally proteins, nucleic acids, or other molecular entities, are indicative of the presence, risk, or progression of cancer in individuals. Their detection and quantification are vital for early diagnosis and monitoring of the disease as mentioned in Table 2. Through electrochemical techniques, a number of these biomarkers can be detected with high specificity and sensitivity.^64^

**Table 2.** Detection and early diagnosis and monitoring of different types of cancers with biomarkers.

Nucleic acid based biomarker detection	Cancer types	References
DNA- DNA binding	Breast cancer	51
RNA-RNA binding	Breast cancer	52
PNA-RNA binding	Cervical cancer	53
DNA aptamer – cells nanoparticles binding	Blood cancer	54
DNA aptamer-antigen binding	Prostate cancer	55
**Cell based biosensors**		
Compound binding ability on cells	Tonsils Prostate cancer Melanoma Hela cell lines	56 57 58
Compond absorption test on cells	Hela cell lines	59
Cell adhesion test	Melanoma Cervix cancer Ovarian cancer	60
**Biomolecule based biosensor**		
Antigen antigen binding	Breast cancer Lung cancer Prostate cancer	4 58
Antigen protein binding	Bone cancer Breast cancer Gastric cancer Lung cancer Liver cancer Colorectal cancer	61 62 60
Protein-DNA binding	Breast cancer	63
Lectin carbohydrate binding	Leukemia cell line	4

#### 5.1.1. Prostate Specific Antigen (PSA)

This biomarker is pivotal for prostate cancer diagnosis. Electrochemical biosensors have proven effective in detecting even low concentrations of PSA.

#### 5.1.2. Carcinoembryonic Antigen (CEA)

Associated with various cancers, including colorectal and gastric cancers, CEA detection using electrochemical methods has been a promising diagnostic tool.

#### 5.1.3. Circulating Tumor DNA (ctDNA)

These are fragments of DNA released by cancer cells into the bloodstream. Their distinct mutations make them detectable targets via electrochemical methods.

#### 5.1.4. MicroRNAs (miRNAs)

Playing a role in cancer progression, these small non-coding RNAs have been the focus of electrochemical detection due to their differential expression in cancer cells.

### 5.2. Examples of recent studies and their findings

In a recent study, a biosensor integrated with gold nanoparticles was employed to detect prostate-specific antigen (PSA), a critical biomarker for prostate cancer. This innovative approach demonstrated exceptional sensitivity, indicating its significant potential for early detection and improved prognosis in prostate cancer patients. By enhancing the signal amplification properties through gold nanoparticles, this method highlights the advancement in biosensor technologies aimed at cancer diagnostics. Another pioneering application involves the use of graphene oxide electrodes for detecting specific microRNAs (miRNAs) associated with breast cancer. This technique has shown remarkable efficiency in identifying miRNA traces even at early stages of the disease. The integration of graphene oxide not only improved the sensor’s conductivity but also enhanced its specificity, marking a substantial step forward in noninvasive diagnostic methods.^10^

A groundbreaking study targeting colorectal cancer developed a multi-target biosensor designed to simultaneously detect carcinoembryonic antigen (CEA) and specific miRNAs linked to the disease. This dual-target capability offers a more comprehensive diagnostic approach, enabling the detection of multiple biomarkers in a single assay. Such a holistic strategy not only enhances diagnostic precision but also provides a deeper insight into the complex biological pathways involved in colorectal cancer. By combining multiple biomarkers, this method paves the way for more personalized and effective cancer management strategies. These advancements collectively underscore the transformative potential of biosensor technologies in revolutionizing cancer diagnostics. They demonstrate the ability to achieve earlier detection, greater accuracy, and a more comprehensive understanding of disease states, thereby contributing to improved patient outcomes and advancing the field of precision medicine.

### 5.3. Innovations in electrode materials and design for enhanced sensitivity

The performance of electrochemical biosensors is greatly influenced by the composition and design of the electrode:

#### 5.3.1. Nanomaterial integration

Gold nanoparticles, carbon nanotubes, and graphene are among materials that, when integrated into electrodes, can augment the available surface area, thus enabling more binding sites and amplifying the resultant signal.^65^

#### 5.3.2. Molecularly Imprinted Polymers (MIPs)

These synthetic polymers, tailored for specific molecule recognition, boost the biosensor’s selectivity when incorporated into the electrode.

#### 5.3.3. Micro and nano-electrode arrays

Through these arrays, the effective electrode surface area is increased, permitting simultaneous multi-biomarker detection. Such designs also improve the signal-to-noise ratio, delivering clearer and more accurate readings.

#### 5.3.4. Hybrid electrode materials

Employing a combination of materials, such as integrating gold nanoparticles with graphene layers, optimizes the benefits of each material. These hybrid designs often exhibit enhanced sensitivity and reduced response times.^55,57,66^ In conclusion, the landscape of electrochemical detection of cancer biomarkers is progressing rapidly. Driven by both the expanding knowledge of cancer biology and breakthroughs in electrode design and materials, these advances pave the way for more efficient and early diagnostic tools, with the potential to revolutionize cancer care.

## 6. Challenges and limitations

### 6.1. Potential interferences in biological samples

Biological samples, like blood or tissue extracts, are complex matrices filled with a plethora of compounds. When analyzing for specific cancer biomarkers, other molecules can potentially interfere with the readings. Electroactive substances, including uric acid, ascorbic acid, and other metabolites, might produce signals at the same or nearby potentials as the target analyte, leading to false positives or inaccurate quantification. Additionally, proteins and other macromolecules can adsorb onto the electrode surface, causing fouling and affecting the sensor’s performance.^67^

### 6.2. Stability and repeatability of the sensors

For an electrochemical sensor to be effective in a clinical setting, it must produce consistent results over multiple tests and maintain its functionality over time. However, challenges arise in maintaining the stability and repeatability of these sensors. The biological recognition elements, such as enzymes or antibodies, might degrade or denature over time, especially if exposed to non-optimal temperatures or pH levels. This degradation impacts the sensor’s sensitivity and specificity. Additionally, sensor-to-sensor variations can sometimes lead to slight differences in readings, questioning the repeatability of results.^68^

### 6.3. Issues with scaling up and commercialization

Translating a prototype biosensor from the research lab to mass production poses several challenges. The manufacturing process must retain the sensor’s sensitivity and specificity while being cost-effective. Often, the delicate biological components of the sensor might not fare well in industrial-scale processes, leading to reduced sensor performance. Commercialization also involves navigating regulatory approvals, which demand rigorous testing and validation, extending the time-to-market. Furthermore, the final product must be user-friendly and robust enough for real-world applications, factors not always considered in initial research designs.^69^

### 6.4. Necessity for validation with standard diagnostic methods

Before any new diagnostic tool, including electrochemical sensors, is adopted broadly, it must be thoroughly validated against established diagnostic methods. This ensures that the new method is at least as effective, if not better, than current standards. However, this comparison can be challenging. Established methods have undergone years or even decades of optimization and have set benchmarks in terms of sensitivity, specificity, and reliability. Electrochemical sensors, while promising, must match or surpass these benchmarks. Additionally, integrating these sensors into current medical workflows and ensuring healthcare professionals are trained and comfortable with this technology is another hurdle to its widespread adoption. In summary, while electrochemical methods for cancer diagnosis are promising and have shown significant advances, they are not without their challenges. Addressing these limitations is crucial for the future success and integration of these tools into mainstream medical practice.^70^

## 7. Future perspectives

### 7.1. Potential for integration with other technologies

The integration of electrochemical methods with other advanced technologies is set to revolutionize cancer diagnostics. Nanotechnology, for instance, offers immense potential. Nanoparticles, with their unique size-dependent properties, can enhance sensitivity and selectivity of sensors.^71^ Gold nanoparticles, carbon nanotubes, and quantum dots have already shown promising results in amplifying electrochemical signals and providing platforms for multi-target detection. Additionally, microfluidics offers a compact, controlled environment for sample processing and detection, streamlining the diagnostic process. Integrating electrochemical sensors with microfluidic chips can lead to lab-on-a-chip devices, making point-of-care diagnostics more efficient and accessible.^72^

### 7.2. Evolution of wearable sensors for continuous monitoring

The future of healthcare leans toward personalized and continuous monitoring. Wearable electrochemical sensors, akin to glucose monitors for diabetics, could become the norm for tracking cancer biomarkers. These devices could offer real-time insights into a patient’s health, allowing for timely interventions and monitoring the effectiveness of treatments. The miniaturization of sensors, combined with advances in materials that are biocompatible and resilient against the challenges posed by the human body, are paving the way for this evolution.^73^

### 7.3. Scope for automation and artificial intelligence in data interpretation

With the influx of data from advanced diagnostic methods, automation becomes paramount to process and interpret the results efficiently. Automated platforms can handle sample preparation, detection, and even preliminary analysis, reducing human error and increasing throughput. Furthermore, artificial intelligence (AI) has the potential to revolutionize data interpretation. Machine learning algorithms can identify patterns and correlations that might be too subtle or complex for human analysts. As these algorithms are fed more data and trained further, their accuracy and predictive capabilities can surpass traditional analysis methods. In the context of electrochemical cancer diagnosis, AI can help in early detection by identifying minute changes in biomarker levels, potentially even before they reach clinically significant thresholds.^74^

## 8. Conclusion

Electrochemical methods have emerged as a transformative approach in cancer diagnostics, offering an impressive combination of sensitivity, specificity, and adaptability for miniaturization. By enabling the rapid and direct detection of cancer biomarkers, these techniques hold immense promise for early diagnosis – a critical determinant of improved patient outcomes and survival rates. This review has highlighted the potential of integrating electrochemical biosensors with cutting-edge advancements in nanotechnology, microfluidics, and artificial intelligence. Together, these innovations present a futuristic vision of precision healthcare, where real-time and continuous monitoring of disease biomarkers could become a standard practice.

Nevertheless, like any emerging technology, electrochemical methods face a range of challenges. Complex biological samples can introduce interferences that affect the accuracy of results, while issues such as long-term stability, scalability, and commercial viability require further attention. Addressing these challenges will necessitate concerted efforts and interdisciplinary collaborations among chemists, biologists, engineers, and data scientists. Such partnerships are essential for designing the next generation of biosensors, capable of overcoming existing limitations and advancing the field of oncology diagnostics.

In conclusion, while electrochemical methods have already demonstrated remarkable potential in cancer diagnostics, their journey is still in its early stages. Continued research and innovation are pivotal to unlocking their full capabilities, paving the way for a new era of cancer care. As these methods evolve, they are poised to not only revolutionize early detection but also redefine personalized medicine, ensuring that today’s technological advancements translate into tomorrow’s life-saving solutions.

## Data Availability

All data generated or analyzed during this study are included in this published article.
